# Modelling socioeconomic determinants for cultivation and *in-situ* conservation of *Vitex doniana* Sweet (Black plum), a wild harvested economic plant in Benin

**DOI:** 10.1186/s13002-015-0017-3

**Published:** 2015-04-30

**Authors:** Sognigbe N’Danikou, Enoch G Achigan-Dako, Dedeou A Tchokponhoue, Chaldia OA Agossou, Carlos A Houdegbe, Raymond S Vodouhe, Adam Ahanchede

**Affiliations:** Horticulture and Genetics Unit, Faculty of Agronomic Sciences, University of Abomey-Calavi, BP: 2549 Abomey-Calavi, Republic of Benin; Bioversity International, West and Central Africa office, 08 BP 0932 Cotonou, Republic of Benin

**Keywords:** Active management, Sustainable utilization, Local knowledge, Domestication, Tree models, Benin

## Abstract

**Background:**

Cultivation is the most appropriate management option when both demand and harvesting of wild plant species increase beyond natural production levels. In the current study we made the assumption that, besides the intrinsic biological and ecological characteristics of the species, the decision to cultivate and/or to conserve an overharvested wild plant species is triggered by the socioeconomic factors such as land tenure and size, origin of respondents, gender, and users’ knowledge of the plant phenology.

**Methods:**

We carried out semi-structured interviews with 178 informants involved in *V. doniana* exploitation. The data collected were related to socio-demographic characteristics of informants’ household situation, knowledge of the biology and propagation of the species, willingness to cultivate the species, in-situ maintenance of populations, and costs associated with management of the species. According to data types we used Student’s t, Spearman correlation, Kruskal-Wallis, Fisher’s exact and χ2 tests to test the effects of land tenure, origin of respondents, gender and users’ knowledge of plant phenology on the decision making process. Conditional inference tree models and generalized additive models were also used to identify variables which were significantly determinant in the decision to cultivate and/or to conserve the species in-situ.

**Results:**

We found that men were more willing to cultivate the species than women and this is conditioned by land area available. The willingness to conserve the species *in-situ* depends mainly on the total land area available, the number of trees within the landscape, accessibility of the trees, land tenure, gender, location, seedling cost, and trade-off cost for conservation. People who offered more than one US dollar to acquire a seedling of *V. doniana,* landowners, and those who own a total land area in excess of 6.5 ha were most willing to conserve the species *in-situ*.

**Conclusions:**

From our findings we conclude that future management and conservation initiatives for *V. doniana* should first target specific user groups for sustainable exploitation of the species. Also, the Cultivation Opportunity Ratio is an important indicator for quick determination of the likelihood of farmers to engage into cultivation and conservation of the species.

## Background

The decision to conserve wild-harvested species is based on factors that are presumed to be community and resource specific. When the value of a non-wood forest product (NWFP) and quantities harvested are both high, it will be under high risk of local extinction, and cultivation is the most effective management option to sustain both the species and livelihoods of the different user groups [1]. The continuous selection and cultivation of variants of useful species to humans, most often, result in domesticated crops [2,3]. Gepts *et al.* [4] indicated that the increase in human demand represents new push for domestication. The main advantages of domesticating intensively wild-harvested edible plant species include: (a) enhanced local livelihoods and food security, (b) increased supply of market commodities and maintenance of food diversity, and (c) increased ecosystem services and resilience. Overall, the economic, social and environmental rewards of domestication are obvious, and domesticated plants should repay better human investments than collection of resources from the wild [5]. For Clement [6] plant domestication is a co-evolutionary process whereby humans select phenotypes of most utility to them and bring them into cultivation. Domestication implies a process in which wild plants are taken from their natural habitats and grown in cultivation systems where growth conditions can be manipulated to enhance production of the desired product (e.g. fruits, leaves, roots, seeds). In simple terms it is the human-mediated adaptation of the wild plant to cultivation in plantation or farmlands [5]. This involves socioeconomic and biophysical processes [7], which lead to both changes to human culture and plant phenology, and begins when species become objects of ownership, inheritance, purchase and exchange [8]. These processes put local communities at the centre of the domestication process, and Clement [6] noted that “*for plant domestication to take place, there must be selection and management*”. Selection of candidates at species and variety levels begins with local communities and is often continued with support of formal breeding and genetic improvement by science. It is important to indicate that domestication is a gradual transition which starts from wild-harvesting through to pre-agriculture (selective harvesting, sparing, etc.), proto-agriculture or incipient domestication (sowing, transplanting, irrigation, etc.), and full domestication characterized by small-scale and/or large-scale agriculture [4]. Success of domestication relies on management of domesticates, which is carried out by farmers. In this context, the recruitment of potential growers and users and their engagement in large scale production of the domesticated species is an important question which needs to be figured out [4].

From the biological point of view, domestication is a continuous endeavour, the result of intensive selection and cultivation of variants with useful traits to humans, with no clear demarcated end points, whereby new phenotypes are created and continuously improved based on new social and economic needs [2,3,6,9]. It then appears that cultivation is the ground for domestication of useful plants to humans. Therefore, in this paper we evaluated the motives for cultivation of wild-harvested economic plants.

Although the scope of ethnobotanical studies has expanded and now covers a wide range of topics related to human - plant interactions and ably demonstrated the importance of wild harvesting to livelihoods of the poor, there is now need to move further to provide insights and guidance for conservation professionals to enhance the sustainable use of highly valued species. For the success of conservation programmes designed by natural resource managers, there is need to understand and integrate drivers of active engagement of local communities, individually and collectively. From the literature, there is very limited knowledge of factors affecting people’s decisions related to plant selection for cultivation [10-12]. Thus, several variables remained unknown for predicting the success or failure of conservation initiatives aimed at bringing wild plants into cultivation. Here, we used *Vitex doniana* Sweet (Verbenaceae) as a case study to investigate and pinpoint local drivers that favour both active cultivation and conservation of wild populations of the species by local communities.

*Vitex doniana* (black plum) is one of the most important wild-harvested multipurpose trees and leafy vegetables of tropical Africa [13-15] particularly in Benin (where it is sold in almost all markets [12,16]), Burkina Faso [17] and Nigeria [18,19] to provide income and support livelihoods of many households. It is one of the most widespread of the 150 species of the *Vitex* genus, with several uses [14,20]. *Vitex doniana* is found in savanna woodlands and gallery forests, and is also indicated to be present in about 50% of traditional agroforestry systems in southern Benin [21], where it is just spared during land clearance [12,22,23]. The species is deciduous and its leaves renewed annually, during the dry season. Intensive harvesting occurs after bush burning associated with farm clearance, with the development of new shoots that are used as vegetable. Leaves, bark and roots also have many applications in indigenous medicine [20]. The fruit is eaten raw or used for making candies, jams and syrups [18,19]. However, the species is currently perceived as the most threatened of edible wild-harvested plants by local communities in Benin [12,24]. Field inventories indicated that the natural populations are continuously eroded and densities of trees and regeneration are becoming low in natural habitats [16,22-24]. Agossou [22], N’Danikou [23] and N’Danikou *et al.* [12] reported that harvesting practices represent the most important threat to the species, contrasting with Oumorou *et al.* [24]’s findings. In fact, harvesting of *V. doniana* vegetable by local communities mainly consists in removing all the new shoots, most often with twigs. Furthermore, some harvesters just cut the whole tree down to make harvesting easier [22,23]. These forms of harvesting reduce fruit production, and subsequently sexual propagation. Meanwhile, the suckers that regenerate from root stock are subject to bush fire in the natural habitat, jeopardizing the natural regeneration of the species. Thus, threat is mainly attributed to overharvesting of wild populations and habitat loss. Therefore, understanding and considering the complex interplay of local valuation and perception of threats would give an important ground for policy and decision making that will sustain the use of the species and also the livelihoods of various user groups.

This situation triggers our study knowing that *V. doniana* is under high harvesting pressure and belongs to a complex market chain [12,13,16], with increased price (authors’ personal observation) whereas natural stands are subjected to private ownership [22]. In this condition which socioeconomic and cultural factors would favour cultivation of *V. doniana*? Is gender an important issue for the success of cultivation? How the access to resources and local knowledge shape the decision-making to cultivate and/or conserve *V. doniana in-situ*?

To answer these questions, we based this investigation on the assumption that, besides the intrinsic biological and ecological characteristics of the species, the decision to cultivate and/or to conserve an overharvested wild plant species is determined by the socioeconomic factors such as land tenure and size, origin of respondents, gender and users’ knowledge of the plant phenology. In fact, in Africa and particularly in Benin, there are societal rules and other factors that affect farmers’ decision to manage useful plants. These factors include gender, age, occupation, wealth status, importance of wild-harvesting in household food security, scarcity of resources, and land holding status [10,11,25,26]. For instance, male farmers and young people were more likely to accept production of non-wood forest products in their farms in Nigeria, because men are more likely to invest in the future than women [10], and this may be because women are uncertain about the security of their tenure due to possible change in their marital status [26]. Also, generally the local customary laws do put restrictions on plantation of perennial crops, particularly tree species which a migrant cannot grow on a rented or borrowed land, unless the land is purchased. This is simply because trees are used to mark human presence and dating land occupation. It is also shown that farmers’ knowledge of the reproductive biology, including phenology, of species is taken into account for selecting candidate species for conservation [3,12].

## Materials and methods

### Study area

**Figure 1:**
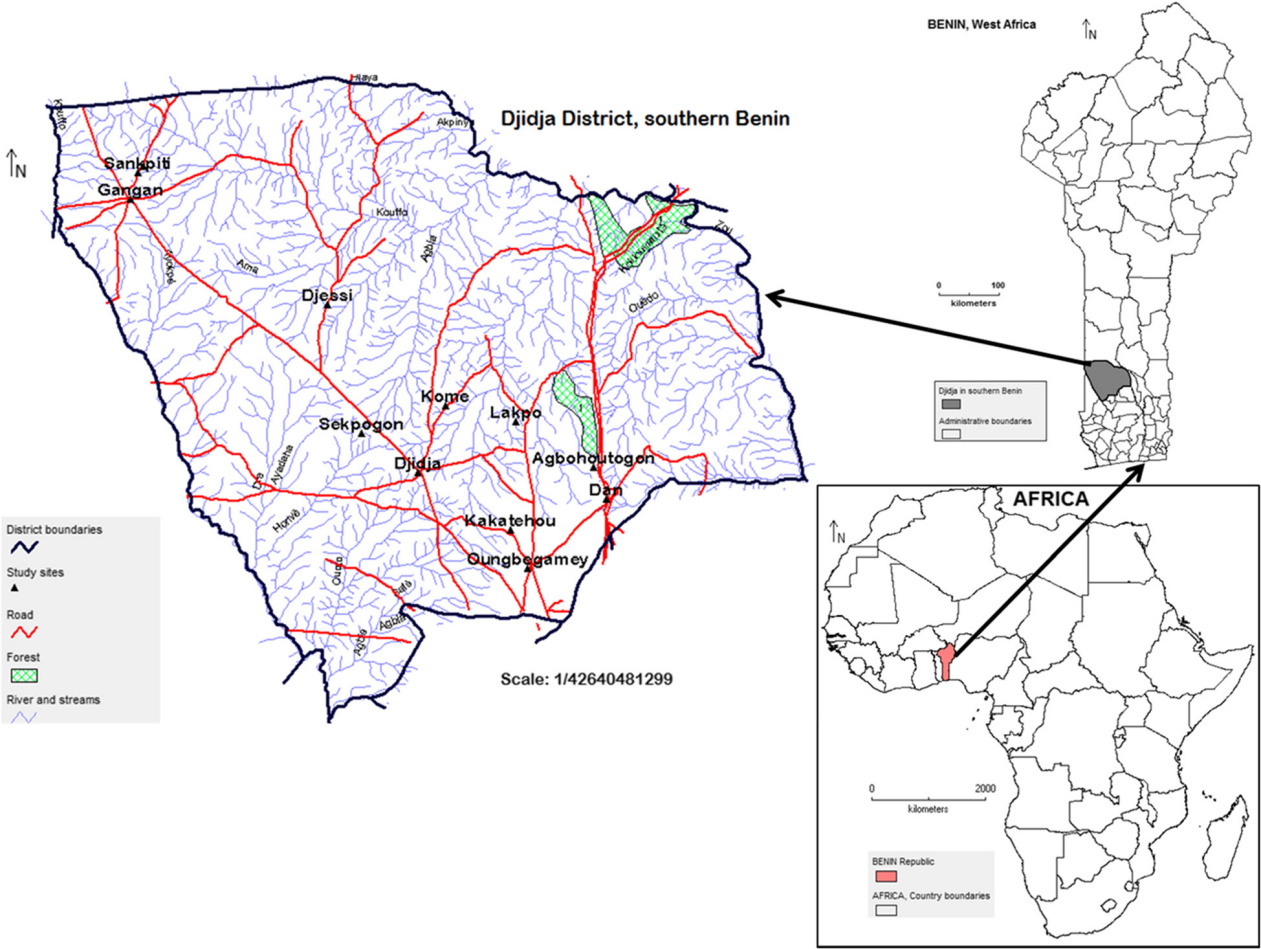
Study area showing surveyed villages in Djidja Ditrict, southern Benin.

This study was carried out in Djidja municipality (Figure 1), one of the most important areas where *V. doniana* is harvested and sold. The species is a highly valued one which is under high human pressure in this area [12,22]. Djidja is the biggest municipality in size (out of nine) in the Zou region (41.66%) in southern Benin. According to the population census by DED-INSAE [27], Djidja holds the second highest population (84,590 inhabitants), representing 14.50% in the entire population of the Zou region. About 78.00% of the district population live in rural areas. The study area is located on flat land at around 250 m altitude in the Sudano-Guinean transition zone. This is a transitional zone between the Guinean forests and the Sudanian woodlands and savannas, and is characterized by a vegetation mosaic of forest islands, gallery forests, and savannas. The annual rainfall varies from 900 to 1200 mm with two rainy seasons (March to July and September to October). Soils are predominantly hydromorphic and sandy-clay. The natural vegetation is made up of savanna woodlands, fallows, and a mosaic of secondary and gallery forests. This landscape provides a range of forest resources from which many edible wild plants are collected, including black plum. However, the natural vegetation is being depleted by land clearance for agriculture, logging for charcoal production, and grazing [12]. As presented below, a total of 12 villages were investigated in this municipality.

### Selection of study sites

Prior to selecting survey villages a market investigation was conducted in local markets in Bohicon and Djidja municipalities which are the major townships where *V. doniana* vegetable is sold. In Bohicon where the regional market is located, twelve vegetable sellers were randomly selected and interviewed. This market survey helped establish the origin of products, the abundance periods and villages supplying the regional market in Bohicon. The interviewees ranked the supplier villages according to the importance of their contributions to the supply. The ranking exercise revealed that the highest share of supply comes from Djidja municipality. In Djidja, a second market investigation was undertaken in five local markets. A total of 42 vendors of black plum products (fruits and/or vegetable) at the local markets were randomly interviewed on the most important supplier villages. Interviewees ranked villages according to the importance of primary collection and sale of the target products. The top 12 villages with higher contribution to the supply were targeted for final survey.

### Informants sampling and data collection

A total of 178 key informants (persons having knowledge of uses and users of *V. doniana* in their community) were identified for interviews using a snowball sampling approach. The number of key informants was deemed representative when a saturation limit was attained. This saturation limit was defined as the point at which there are no new names of key informants being mentioned by the last interviewee. At this point, the plot of the number of key informants becomes asymptotic with the addition of any new interviewee. The data collected include three categories of information: a) the socio-demographic characteristics of informants (gender, age, ethnic background, marital status, size of household, land ownership, origin, education of the head of household, occupation and revenue sources, social group membership, social responsibilities, and experience with management of wild species), b) ranking of the five most important and valued tree species to the respondent, and c) informant’s knowledge of black plum (ownership status, uses and use frequency, knowledge of the phenology, threats and drivers, individual conservation initiatives, trade-off and cultivation opportunity costs and ratio, and willingness to cultivate and to maintain the species *in-situ*).

We obtained the permission from the chief of each village involved in the study before interviews were conducted, following the ethical guidelines of the International Society of Ethnobiology [28]. Participants were selected and included in the study after obtaining their verbal prior informed consent.

No ethical approval was needed for this study.

### Data analysis

#### Determination of the importance and use value of Vitex doniana in the surveyed communities

Before testing the research hypotheses, we first determined the relative importance of *V. doniana* within the diversity of useful local trees, against a number of socioeconomic characteristics. Based on value scores attributed by each respondent for the five most important tree species to them (55 species mentioned in all), we calculated the Species Use Value (SUV) for each species as the sum of the species value for all 178 respondents as:

$$ SU{V}_i={\displaystyle \sum_{j=1}^n{S}_{ij}} $$

with *S*_*ij*_ being the score given to the species *i* by the informant *j*.

Ranks were converted into scores and SUV calculated using the method by Lawrence *et al.* [29]. The species that was ranked first by a respondent received the score 5 whereas the fifth received the score 1. A species which was not mentioned by a given informant was given the score zero. The SUV of each species was disaggregated by gender, origin and schooling of respondents.

We also determined the Relative Use Value (RUV) of *V. doniana* for each category of informant (for e.g. farmers versus vegetable sellers). This helped identify user groups with specific knowledge on the use of the species and the relative importance to them.

$$ RU{V}_k=\frac{1}{n}{\displaystyle \sum_{j=1}^n{S}_{kj},} $$

adapted from Phillips *et al.* [30].

S_*kj*_ is the informant *j* of user group *k*’s use value for the species; *n* is the total number of informants in the user group *k*.

To reveal a minimum consensus on the use of the species in the community only species cited by at least two respondents were included in further analyses. We used Student’s *t*-test to examine the influence of gender and origin and Kruskall-Wallis’ statistic to test the influence of schooling on the perceived value attributed to *V. doniana.* We also determined the Informant Agreement Ratio (IAR) following Bakwaye *et al.* [31] as follow:

$$ IA{R}_i=\frac{n{r}_i-n{a}_i}{n{r}_i-1} $$

where nr_*i*_ is the total number of citations recorded for a given use category *i*; and na_*i*_ the total number of different plant parts that are employed in this specific use category *i*. This index measures if there are specific plant parts employed for particular uses. Five different use categories were identified, viz. food, income, medicine, construction, and others (handicraft, ink, stools).

#### Test of research hypothesis using classical non-parametric tests

We used Fisher’s exact test to test if resource management depends on socioeconomic variables such as tree ownership, origin of respondent, and accessibility to resources. The relationships respectively between gender, land tenure and land holding, schooling and literacy, origin, participation in social organizations, level of income earned from *V. doniana* sales, technical training and management of *V. doniana* were evaluated using Spearman’s correlation tests.

As cultivation of the species was not yet evident in Djidja, we assessed the economic value and respondent’s willingness to cultivate and also to conserve the species based on five parameters, namely the Cultivation Opportunity Ratio (COR), the Land Allocation Ratio (LAR), the Trade-off Value (TOV) which represents the minimum amount the informant is ready to accept for the maintenance of one “*Cantin*” (equivalent 400 m^2^) of the species, the willingness to conserve the species in the wild, and the willingness to experiment with cultivation of the species. These are new parameters that we introduced to determine socioeconomic drivers for plant cultivation by local communities. COR and LAR are calculated as follow:

$$ COR=\frac{S_p-{A}_c}{S_p}*100\kern0.5em ;\kern0.5em  and\kern0.5em  LAR=\frac{S_{\max }}{S_t}*100 $$

*S*_*p*_ is the estimated price at which the respondent would sell a seedling of black plum, *A*_*c*_ is the estimated cost that the respondent would be willing to pay to acquire a seedling of black plum, *S*_*max*_ is the maximum size of land an informant is ready to allocate to black plum within his/her farm and *S*_*t*_ the total land size s/he holds. We tested differences in COR, LAR, and TOV using Mann–Whitney and Kruskal-Wallis’ tests. Relationships between household socioeconomic characteristics and willingness to maintain the species in its natural habitat (*in-situ* conservation) and/or to invest in black plum cultivation were tested using Chi-square or Fisher exact tests as appropriate.

#### Test of research hypotheses using statistical models

Due to the large number of explanatory variables (69 in total), we used the conditional inference tree model to select variables with most significant effects on the decision to cultivate and/or to conserve the species *in-situ*. The tree model is appropriate in cases where there is large number of explanatory variables and these can be a mix of continuous and categorical variables [32]. The tree-based models have a number of advantages in estimating a non-parametric regression relationship by binary recursive partitioning using conditional distributions. Such models are not based on any kind of distribution assumptions, and most importantly complex interactions with a large number of explanatory variables and non-linear relationships that are difficult to examine with traditional statistical methods can be modelled. Further details on tree models and conditional inference tree-based classification can be found in Hothorn *et al.* [33] and Crawley [32]. Further analyses of the relationships between the selected variables with the response variables (willingness to cultivate and willingness to conserve wild populations *in-situ*) were tested with generalized additive models (GAMs) which are appropriate for binary responses with at least one continuous explanatory variable.

### Definition of terms

#### *In-situ* conservation

In Article 2 of the Convention on Biological Diversity (CBD) *in-situ* conservation is defined as “*the conservation of ecosystems and natural habitats and the maintenance and recovery of viable populations of species in their natural surroundings and, in the case of domesticated or cultivated species, in the surroundings where they have developed their distinctive properties*” [34]. On-farm conservation of useful species by farmers has been recognized as an *in-situ* or *circa-situ* conservation strategy [4,35].

#### *Ex-situ* conservation

In the same Article 2 of the CBD, *ex-situ* conservation means “*the conservation of components of biological diversity outside their natural habitats*”. The conservation of germplasms in genebanks is recognized as an *ex-situ* measure as is conservation in botanical gardens [4].

## Results

### Household characteristics of informants

Women represented 60.00% of informants, and the Fon ethnic group dominated the sample (89.00%). Other ethnic groups include, by importance, Agouna, Mahi, Nagot and Adja. *Vitex doniana* resource users were between 12 to 66 years old, and 42.73 ± 16.12 years in average. The sample was characterized by a low proportion (17.98%) who has attended primary school and the main activities were farming, and trade of agricultural products. About 83.00% of respondents are owners of their land (with an average of 12.05 ha per household) whereas those who rent their land represented 16.29% with an average of 4.91 ha/household. Those who were landowners but also rented additional land for farming represented a very small proportion (0.71%). About 38.40% of respondents participated in social groups; the most common of these included self-help groups who provide agricultural product storage, groups formed to undertake sales and processing of agricultural products. Women participated more than men in such groups. Autochthons represented 68.00% of the sample against 32.00% migrants. The size of households greatly varied. Households with less than five members represented 28.65% of the sample; a high proportion of households had between 5–10 members (52.81%). Those with 11–15 members represented 16.85%, and households with more than 15 members represented 1.69%. All the households were involved with black plum.

### Diversity of most valued tree species in the communities

From the individual respondents’ lists of their five most important tree species, we recorded a total of 55 species belonging to 24 families. The determinants of the value given by respondents included the nutritional value, the market value, the medicinal value, the cultural functions and use in construction. The families most often mentioned were Leguminosae-Caesalpinioideae and Anacardiaceae. The species mentioned in the Leguminosae-Caesalpinioideae family included *Daniellia oliveri* (Rolfe) Hutch. & Dalziel, *Afzelia africana* Pers., *Detarium microcarpum* Guill. & Perr., *Bauhinia thonningii* Schum., and *Tamarindus indica* L.; those in the Anacardiaceae included *Anacardium occidentale* L.*, Mangifera indica* L., *Lannea schimperi* (Hochst. ex A.Rich.) Engl., and *Spondias mombin* L. The results also indicated that wild species were dominant (60.00%), especially *V. doniana* and *Parkia biglobosa* (Jacq.) G.Don which were respectively ranked the second and the third species, behind *A. occidentale* which was a cash crop in the survey area. The seven most frequently mentioned species have high nutritional and market values, whereas the next eight species are mainly used in folk medicine and construction (Table 1).

**Table 1:** **Top 15 priority tree species based on species use values (SUV); wild species are in bold**

**Species**	**Instruction level**			**Rank**_**inst**_	**Origin**		**Rank**_**Orig**_	**Gender**		**Rank**_**gend**_
	**No schooling**	**Primary**	**Secondary**		**Indigenous**	**Migrants**		**Men**	**Women**	
*Anacardium occidentale* L.	2.16	1.92	1.50	1	2.40	1.98	1	2.33	2.17	1
***Vitex doniana*** **Sweet**	2.38	1.67	0.25	3	2.15	2.53	2	1.52	2.61	2
***Parkia biglobosa*** **(Jacq.) G.Don**	2.15	1.68	1.25	2	1.94	2.09	3	1.75	2.26	3
*Mangifera indica* L.	1.26	1.27	0.88	4	1.09	1.49	4	0.82	1.44	5
*Elaeis guineensis* Jacq.	1.15	1.00	1.88	5	1.49	0.82	5	1.94	0.69	4
*Tectona grandis* L.f.	1.36	1.03	1.38	6	1.02	0.94	7	1.42	0.84	6
*Citrus sinensis* (L.) Osbeck	1.53	0.48	0.50	7	1.14	1.12	6	1.28	0.97	6
***Pterocarpus erinaceus*** **Poir.**	0.51	1.19	0.00	8	0.70	0.48	8	1.00	0.34	8
***Vitellaria paradoxa*** **C.F.Gaertn.**	0.56	0.58	1.00	9	0.47	0.50	9	0.43	0.53	9
***Pseudocedrela kotschyi*** **(Schweinf.) Harms**	0.24	0.35	0.00	10	0.19	0.25	11	0.09	0.30	11
***Ficus sur*** **Forssk.**	0.28	0.20	0.25	12	0.20	0.24	10	0.03	0.31	12
***Prosopis africana*** **(Guill. & Perr.) Taub.**	0.17	0.38	0.00	11	0.24	0.02	15	0.27	0.16	10
*Azadirachta indica* A.Juss.	0.12	0.33	0.00	13	0.08	0.25	12	0.22	0.12	12
***Daniellia oliveri*** **(Rolfe) Hutch. & Dalziel**	0.11	0.32	0.00	14	0.16	0.18	14	0.18	0.12	14
***Lannea schimperi*** **(Hochst. ex A.Rich.) Engl.**	0.15	0.18	0.00	15	0.15	0.13	13	0.16	0.15	14

We also found that among the wild species *V. doniana* was the most valued. This perception was not affected by whether the informant was indigenous or migrant (t = 0.47; p = 0.64). However, gender (t = 6.48; p < 0.001) and schooling (H = 8.62; p = 0.013) significantly affected the perceived value ascribed to the species. Women valued the species significantly higher (mean SUV = 2.61) than men (mean SUV = 1.52). *Vitex doniana* seemed to be the most valued species by people who have not attended school (SUV = 2.38) and this value decreased with schooling (SUV = 1.67 for primary and SUV = 0.25 for secondary and higher education). Gender, origin and schooling also showed strong and positive correlations with each other (r ≈ 0.99; p < 0.001).

### Variations in *Vitex doniana* use patterns

A total of 34 different uses were recorded for six parts of *V. doniana*, namely fruits, leaves, bark, trunk, branches and roots (Table 2). Those uses were classified into five use categories such as household food consumption, folk medicine, sales, construction material and fuel, and others (stools, handcrafts, ink making). The values of Informant Agreement Ratio (IAR) calculated for each use category revealed a consensus on the use of the different plant parts (Table 3). All plant parts are used but roots are only used in folk medicine. Leaves and fruits are used for human food and also sold in markets to generate income. All plant parts had applications in traditional medicine, except trunk and branches; the most commonly treated ailment being haemorrhoids.

**Table 2:** ***Vitex doniana*** **plant parts and their uses in Djidja district**

**Plant parts used**	**Use categories**	**Uses**
Roots	Folk medicine	Against snake and scorpion bites, haemorrhoids, malaria, ulcers, diabetes, sexual impotence, stomach-ache, wounds, constipation, eye pains, intestine worms, sprain
Bark	Folk medicine	Sterility, haemorrhoids, snake and scorpion bites, intestine worms, ulcers, stomach-ache, diabetes, constipation, malaria, wounds, sexual impotence, eye pain, cough, amenorrhea, dysmenorrhea, strokes
Trunk and branches	Construction and fuel	Fuel wood, charcoal, planks, beam
	Other uses	Drums, stool of the king
Leaves	Food	Household use for consumption as cooked vegetable
	Sales	Sale as cooked vegetable
	Folk medicine	Haemorrhoids, snake bites, mouth candidiasis, whitlow, annulet, malaria, ulcers, sexual impotence, stomach-ache
	Other uses	Ink
Fruits	Food	Direct consumption of fruits, juice making
	Sales	Sale of fruits for direct consumption
	Folk medicine	Haemorrhoids

**Table 3:** ***Vitex doniana*** **major use types and Informant Agreement Ratio (IAR)**

**Use categories**	**Number of use citations (nr)**	**Plant parts involved**	**Number of parts involved (na)**	**IAR**
Construction and fuel	3	Trunk and branches	2	0.500
Food	298	Fruits and leaves	2	0.996
Sales	120	Fruits and leaves	2	0.992
Folk medicine	58	Fruits, leaves, bark, roots	4	0.947
others	11	Trunk, branches, and leaves	3	0.800

The relative use value (RUV) calculated for each user group indicated that traders of black plum products ascribed higher use value (RUV = 3.26) to the species than other stakeholders (Table 4). The average profit from sales of *V. doniana* vegetable during the last season was estimated at XOF 38,100 (US $79.16) per user. The results also indicated that women realized more profit from sales than men (p < 0.001). Although youths realized more profit than teenagers and elderly people the differences were not statistically significant between age groups (Table 5). The values of the coefficient of variation were greater than 1 for all age categories and for women. This indicates a big variability in the profit earned by these groups out of the sale of *V. doniana* products. It is noteworthy to mention that the profit did not follow a normal distribution. Overall, there is an uneven distribution of the profit along the value chain of *V. doniana* (this was examined further in another study and not discussed here).

**Table 4:** **The relative use values (RUV) of** ***Vitex doniana*** **per user group**

**Occupations**	**Total scores**	**n**	**RUV**
Traders of *V. doniana* products and other small businesses	137	42	3.26
Farming only	182	79	2.30
Artisans, forest product businesses	33	16	2.06
Transport, part time farming, singing, traditional healer	20	10	2.00
Other traders of household goods, plus other activities	46	31	1.48

**Table 5:** **Profits from** ***Vitex doniana*** **products sale by different age categories and gender in Djidja district**

**Variable**	**Groups**	**Profit earned**	**Standard deviation**	**Coefficient of variation**	**Significance**
	<20 years	22,787.5	38,399.7	1.7	Age (p = 0.206)
Age	20 – 55 years	41,067.2	41,704.7	1.0	
	>55 years	29,576.9	34,720.8	1.2	
	Men	12,916.7	9,769.4	0.8	Gender (p = 0.000)
Gender	Women	40,290.6	41,268.2	1.0	

### Management of *Vitex doniana*

**Figure 2:**
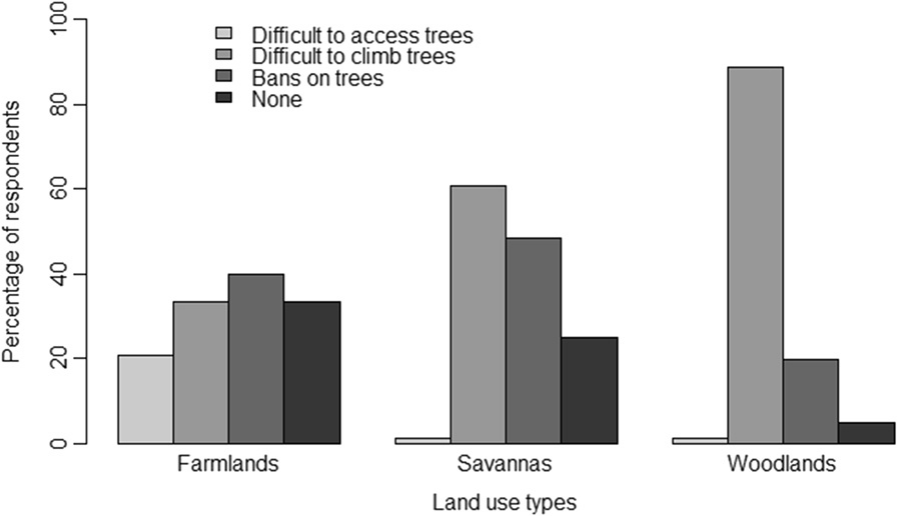
Perception of constraints to *V. doniana* exploitation in different land use types.

*Vitex doniana* is under both individual and collective management in the study area, with collective management being dominant. About 92.00% of informants maintained trees of the species within their farmlands. The species is harvested from different habitats. By decreased order of importance, *V. doniana* is harvested from farms and fallows (100% of informants), savannas (87.00%), and woodlands and gallery forests (83.00%). An important proportion of informants (55.60%) reported that the species was either very scarce or scarce. According to informants three main constraints were encountered during harvesting, namely: difficulties accessing some sites due to thickets, the personal risks of climbing trees for harvesting, and privatization of some trees (Figure 2). Trees occurring in savannas and woodlands were more difficult to climb while privatization was more prominent for trees occurring on farms and fallows. Felling of taller trees to harvest new shoots has become a common practice.

**Figure 3:**
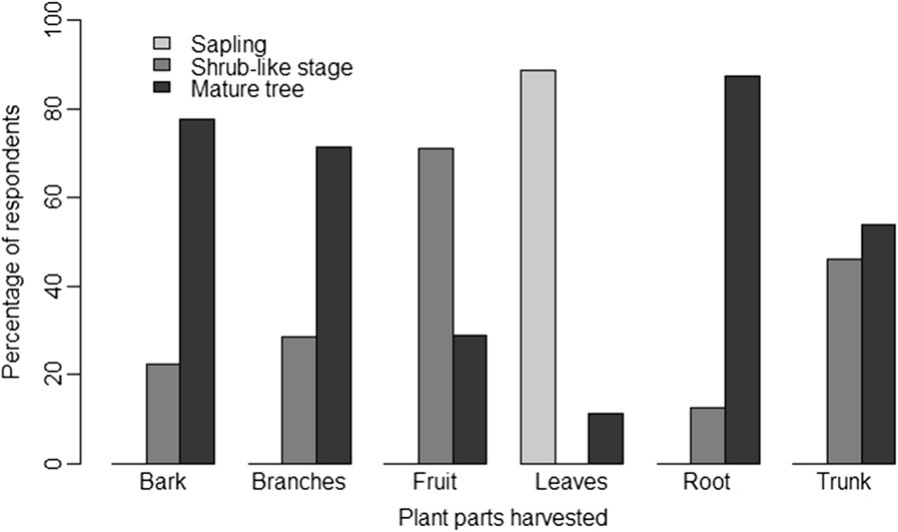
Perception of the minimum growth required before harvesting the different parts of *Vitex doniana.*

The people’s knowledge of species’ phenology was highly variable. For instance, knowledge of the fruiting period was dependent on gender and origin of respondents (p < 0.001). Men (average score = 5.19) had better knowledge of fruiting period than women (average score = 3.51); and autochthones (average score = 5.23) had better knowledge of the fruiting period than migrants (average score = 2). In addition, men had a significantly better knowledge of the period of appearance of new shoots than women (p = 0.02). Land tenure also significantly affected this knowledge (p < 0.001), with the best knowledge of shooting period registered among autochthon landlords. Moreover, schooling seemed to be negatively correlated with knowledge and we reason that attendance at school deprives children of the chance to be in contact with the species, hence elite people in the surveyed communities had less knowledge on the biology compared to those who have not gone to school (p = 0.01). Results also indicated that there was a minimum growth required before a specific plant part can be harvested and the timing of this varies with the plant parts harvested (Figure 3). Except for leaves, harvesting of all other plant parts (e.g. bark, branches, stems, and roots) starts at the earliest at the shrub-like stage. Harvesting of leaves can occur on juvenile subjects, while fruits harvesting occurs only on mature trees.

### Local perception on status and conservation strategies

Most informants (55.00%) considered *V. doniana* populations as declining in the local environment and this perception was equally shared among men and women farmers (p > 0.05). According to our informants, the decline in *V. doniana*’s populations is triggered by human pressure that is characterized by intense harvesting of new shoots, sometimes involving cutting of whole tree that are difficult to climb. The species is also used in charcoal for which mature trees are cut down. About 50.00% of informants reported that cutting of *V. doniana* tree to harvest leaves was the main threat to the species. Informants also reported low regeneration rates which they attributed to low production of fruits and low germination rates (7.92% of informants), natural death of stands, and other drivers such as bushfires (2.97%), agricultural expansion and urbanization (2.97%).

Respondents recognized propagation through seeds (81.47%) as the major route to regenerate the species. Only 6.17% mentioned the possibility of using stem cuttings. However, attempts to propagate the species were very rare and only 5.61% of informants (five men and five women in a sample of 178) have tried to regenerate it. Among respondents, only owners of land (acquired either through inheritance or gift) regenerated the species. Among those who have tried, 30.00% of them experimented regeneration with stem cuttings; the other 70.00% used seeds. The success rate is 33.33% (only one success over three cases reported) for stem cuttings and 57.14% (four successes over seven cases reported) for the regeneration by seeds. When disaggregated by gender, this success rate was 60.00% for women and 40.00% for men. In the cases reported no additional care was provided to the transplanted seedlings or the regenerated plants. Successful results were obtained for seedlings transplanted in the wet season; seedlings were rain-fed. Trees produced by these means were found on-farm and were intensively harvested by their owners. No consideration of selective sowing of seeds or planting of stem cuttings was reported.

### Readiness of communities to engage in cultivation of *Vitex doniana*

#### Exploration of relationships between socioeconomic variables and willingness to actively conserve the species as wild and cultivated resource, using Fisher’s test

In order to better promote cultivation of *V. doniana* in the surveyed communities, we evaluated informants’ willingness to engage in its active conservation (i.e. both planting and caring of the trees on-farm but also maintenance of wild trees *in-situ*). Overall, 76.50% of informants were ready to invest in agroforestry of the species*,* whereas 74.40% said they are willing to maintain woodlots of the species. The Fisher test on individual variables indicated that the willingness to plant the species seemed independent of gender, origin, land ownership, literacy level, schooling, and household consumption or sale of black plum products (p > 0.05). Plant resources management was also independent of social group membership and participation in technical training (p > 0.05). However, the willingness to maintain wild trees *in-situ* was significantly affected by land tenure (p < 0.05). Landowners were the most willing to conserve stands of the species. However, this does not significantly affect the trade-off value (TOV). The TOV was only significantly affected by schooling (p < 0.05). We observed that people reaching secondary and higher formal education reported TOVs more than three times higher (XOF 270,000; equivalent USD 600) to maintain a 400 m^2^ stands of black plum, compared to those who stopped at primary level (XOF 78,000; equivalent USD 173). The average land size that respondents were ready to allocate for *V. doniana* agroforestry is 0.98 ha, representing an average land allocation ratio (LAR) of 8.33%. The LAR was significantly affected by gender (p < 0.05). Women were ready to allocate an average of 23.66% of their land to black plum while men were only willing to allocate an average of 14.04% of their agricultural land to *V. doniana*. However, this did not imply that women allocated more land than men. In absolute terms men’s land holding (18.9 ha in average) is far larger than that of women (5.6 ha in average). These ratios imply that men allocated 2.7 ha while women allocated 1.3 ha.

The *in-situ* conservation cost was not evaluated for 8.42% of the respondents, as they were not able to evaluate the costs related to this. For the other 91.58%, there was no significant difference in the Cultivation Opportunity Ratio (COR) with respect to gender, origin, access, and schooling. However, the COR had significant effect on the willingness to cultivate (Table 6) or to conserve the species *in-situ* (Table 7). Negative COR values indicated the informants were ready to be actively engaged (p < 0.05). The average acquisition cost an informant was ready to pay for a *V. doniana* seedling was disaggregated by negative and positive COR values. These values indicated a great variation among the amounts proposed by users. The negative COR was associated with a high value ascribed to the seedling by the informant in a way that s/he was ready to offer higher price to acquire than s/he would sell his/her own seedlings. Interestingly, informants who were most willing to cultivate or to conserve wild plants of *V. doniana* (negative COR values) offered the highest prices to acquire seedlings. Therefore, the sign of the COR is a good indicator of the willingness to cultivate, but also the willingness to conserve the species *in-situ*. Among those with negative COR, all are willing to cultivate and also willing to conserve, excepted one informant. This was more prominent with other respondents who indicated they “*will never sell seedling of black plum*” due to its importance for them. Those informants were not included in the calculations, as they did not suggest any price. In the case of positive values of COR, there seemed to be additional variables that are taken into account in the decision making.

**Table 6:** **Willingness to cultivate and mean seedling price and acquisition cost (in XOF) for positive and negative cultivation opportunity ratio (COR) value groups, N = 101**

	**Negative COR**		**Positive COR**		**Significance**
Mean ± sd	−1.1 ± 2.1		21.7 ± 82.5		p < 0.05
	cultivation		cultivation		
	Yes	No	Yes	No	
	n = 23	n = 0	n = 61	n = 17	
Selling price	115.2 ± 424.6	-	10,300.0 ± 29,655.6	42,297.1 ± 122,605.6	p < 0.05
Acquisition cost	958.7 ± 1,677.8	-	753.8 ± 1,928.0	370.6 ± 1,197.6	p < 0.05

**Table 7:** **Willingness to conserve species** ***in-situ*** **and mean seedling price and acquisition cost (in XOF) for positive and negative cultivation opportunity ratio (COR) value groups, N = 101**

	**Negative COR**		**Positive COR**		**Significance**
Mean ± sd	−1.1 ± 2.1		21.7 ± 82.5		p < 0.05
	Conservation		Conservation		
	Yes	No	Yes	No	
	n = 22	n = 1	n = 56	n = 22	
Selling price	139.5 ± 465.6	0.0 ± 0	10,879.2 ± 31,640.4	36,702.4 ± 11,0351.1	p < 0.05
Acquisition cost	1,001.1 ± 1704.6	25.0 ± 0	970.0 ± 2140.2	83.6 ± 116.3	p < 0.05

#### Determinants of the willingness to cultivate Vitex doniana, using the conditional inference tree approach

**Figure 4:**
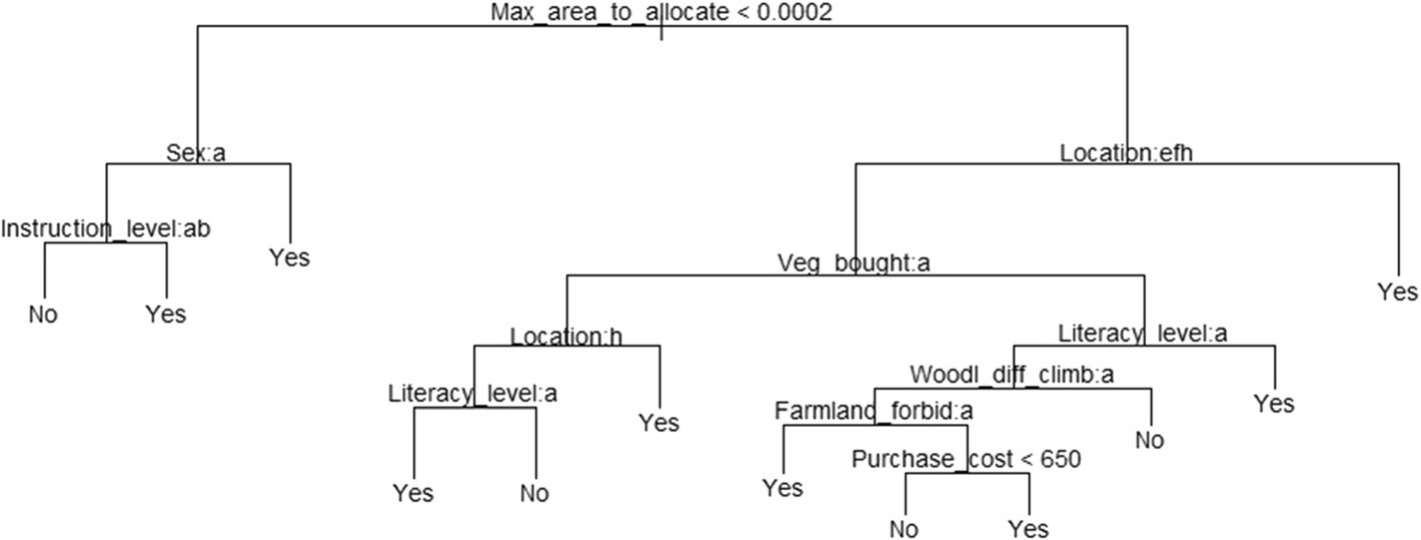
Conditional inference tree showing factors determining the decision of local people to cultivate *Vitex doniana*. The response Yes = the informant is willing to cultivate; and No = informant not likely to cultivate. Sex (a = Female, b = Male); Instruction level, with reference to western education (a = No schooling, b = Primary, c = Secondary and higher education); Literacy level (a = Cannot read nor write in mother tongue “illiterate”, b = Can write and read in mother tongue “literate”); Location (a = Agbohoutogon, b = Dan, c = Djessi, d = Djidja centre, e = Kakatehou, f = Kome, g = Lakpo, h = Oungbega, i = Sekpongon); Veg_bought, i.e. Vegetable bought or purchase of *Vitex doniana* vegetable (a = No, b = Yes); Woodl_diff_climb, i.e. Difficult to climb *Vitex doniana* trees in the woodland (a = No, b = Yes); Famland_forbid, i.e. Harvesting is forbidden in farmland (a = No, b = Yes).

The conditional inference tree classification using all socioeconomic and biological variables (Figure 4) indicated that the maximum land size that people are ready to allocate for the agroforestry of *V. doniana* was the main indicator of readiness to cultivate. Among people whose maximum allocated land was less than 0.0002 ha, gender was key in the decision making process, and men were more (or the most) willing to cultivate. For the women informants in this category, educational level determined the decision. Non-schooled women and school drop-outs were not willing to cultivate *V. doniana*, but those women who have attended secondary or higher education were more willing to cultivate the species.

Among those farmers ready to allocate more than 0.0002 ha, people from Agbohoutogon, Dan, Djessi, Djidja centre, Lakpo, and Sekpongon villages were the most engaged to cultivate. In the other locations such as Kakatehou, Kome, and Oungbega, the type of access to resources (e.g. wild harvesting or purchase) was key in the decision making process. Among people who collect *V. doniana* in the wild, those from Kakatehou and Kome were ready to cultivate, without condition. Among this category, literacy level plays a role in the decision-making process in Oungbega and illiterate people (who cannot read and write their mother tongue) were most likely to cultivate the species; the literate were not. Among those who can only access the resource through purchasing, either for trading or for household consumption, in Kakatehou, Kome, and Oungbega; literacy level was also a determinant, and literate people were most willing to cultivate. Among the illiterate, perception of the hardship endured to climb trees to collect the leaves was crucial in the decision making process, and surprisingly those who find it difficult to climb trees in woodlands were not willing to cultivate the species. Among those who did not perceive it difficult to climb trees in woodlands, the existence or not of protection mechanisms of trees in private lands (farmlands) plays a role in the decision to cultivate. In fact, in farmlands where there is no ban on trees, people were more likely to cultivate. In communities where trees occurring on farmlands were private resources, the maximum amount the informant is ready to pay to acquire a seedling of the species was the best indicator of the willingness to cultivate with highest levels among those proposing over XOF 650 (USD 1.44) per seedling. This is well in line with the results presented in Table 6.

#### Determinants for the conservation of wild trees of Vitex doniana in-situ, using the conditional inference tree approach

**Figure 5:**
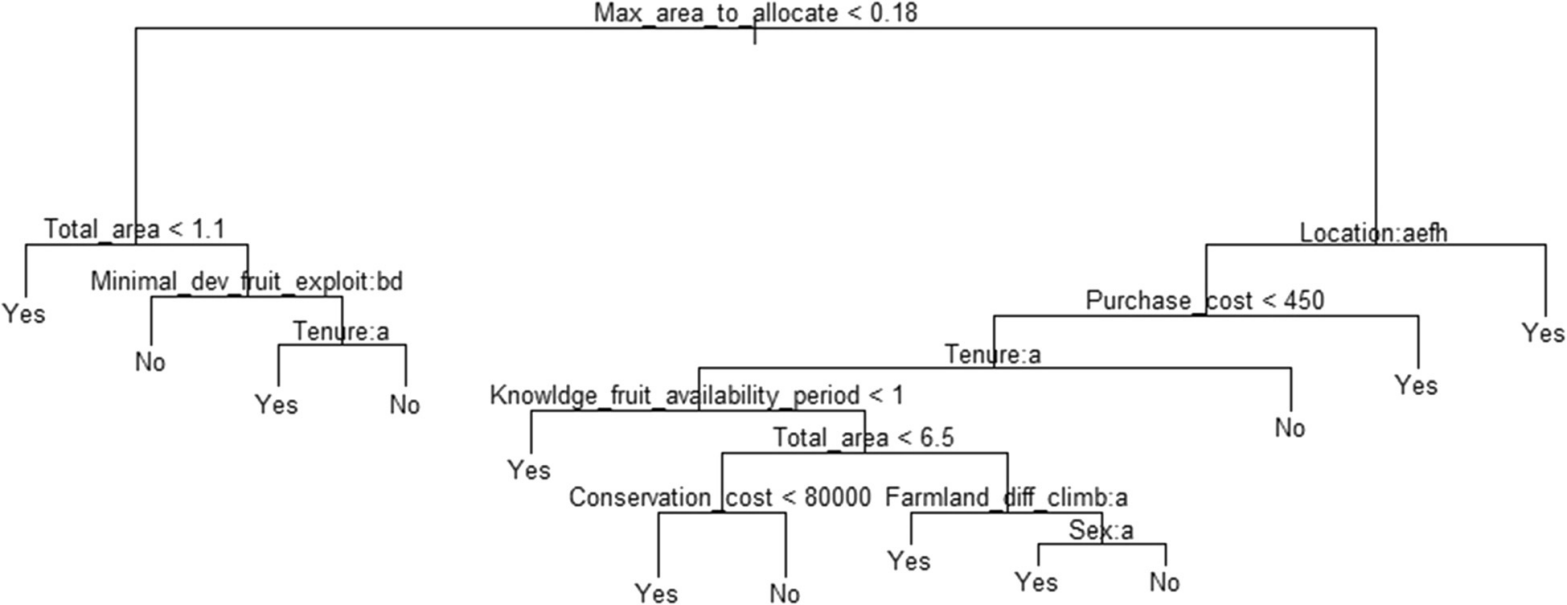
Conditional inference tree showing factors determining the decision to conserve wild trees of *Vitex doniana in-situ.* The response Yes = the informant is willing to conserve the species *in-situ*; and No = informant not likely to conserve the species *in-situ*. Minimal_dev_fruit_exploit, e.i. The minimal plant developmental stage required for exploitation of fruits (a = Juvenile stage, b = Fruits not used, c = Shrub-like stage, d = Mature tree stage); Tenure (a = Land owner, b = Tenant of land); Location (a = Agbohoutogon, b = Dan, c = Djessi, d = Djidja centre, e = Kakatehou, f = Kome, g = Lakpo, h = Oungbega, i = Sekpongon); Farmland_diff_climb, i.e. Difficult to climb *Vitex doniana* trees in the farmland (a = No, b = Yes); Sex (a = Female, b = Male).

The conditional inference tree (Figure 5) indicated that the maximum area people were ready to allocate was a main factor in respondents’ willingness to conserve the species *in-situ*. For people allocating less than 0.18 ha of land to *V. doniana*, the total land available was key to the decision, and people with less than 1.1 ha of total available land were the most likely to conserve the species. Among those with more than 1.1 ha of land, availability of *V. doniana* trees (measured through abundance of mature trees for harvest) was determinant to the decision making process, and people who have easy access to trees were not likely to conserve wild populations. Among those with limited access to the resource (assessed as high pressure on immature trees), tenure has a bearing on the decision and only landowners were likely to conserve, land tenants were not.

For people who were ready to allocate more than 0.18 ha to the species, the decision to conserve depended mainly on location, with people from Dan, Djessi, Djidja centre, Lakpo, and Sekpongon villages more engaged into for its conservation. For other respondents from Agbohoutogon, Kakatehou, Kome, and Oungbega, the perceived cost of one seedling was key to the decision. People who were ready to pay more than XOF 450 (1 USD) were most engaged to conserve the species *in-situ*, supporting the results in Table 7. Among informants who proposed less than 1 USD, tenure system has to be taken into account, with tenants not likely to conserve. Among landowners of this category, main variables used in the decision making process included knowledge of plant phenology (fruiting period), the total land available, the trade-off value (TOV) or conservation cost, the perceived difficulties in climbing trees to harvest products and gender. In fact, among those farmers with less than 6.5 ha of total available land, people who requested a TOV below XOF 80,000 (USD 177) were likely to conserve, while those who requested more than USD 177 were not. Among the farmers with more than 6.5 ha of total land, people who perceived it less difficult to harvest the trees occurring within farmlands were more likely to conserve. Among those who find it difficult to harvest trees on their farm, only women were ready to conserve the species *in-situ*, men were not.

## Discussion

### Valuation of tree resources in the surveyed area

Findings indicated that wild species represented the major part of the edible tree resources diversity in the twelve surveyed communities, meaning that local people still rely to a great extent on nature for their livelihoods. Out of the top 15 most valued woody plants, eleven have a high market value and species such as *A. occidentale, V. doniana*, and *P. biglobosa* combined high market and nutritional values. *Vitex doniana* stood out as the most important wild tree species followed by *P. biglobosa*, with both falling just behind *A. occidentale* which is an important cash crop in the surveyed communities. This result is consistent with findings by several authors who investigated the use of plant diversity in rural livelihoods in the West African sub-region [16,36-38]. *Parkia biglobosa* and *V. doniana* were already reported among the most valued NWFP resources in southern Benin [12], in Burkina Faso and Cameroon [39]. The two species are almost always used together in local cuisine where the local mustard “*afintin*” processed from seeds of *P. biglobosa* is used as seasoning for cooking leaves of *V. doniana* into a much appreciated popular vegetable dish in the region, known as “*fonman*”. Therefore, the current results call for a close attention for safeguarding and valuing these species.

Cultivation appears to be the best management option to sustain both species and livelihoods when the value of a non-wood forest product (NWFP) and quantities harvested are high [1]. This opinion was also backed by Kupzow [5] who supported that plant cultivation is most stimulated by economic demand and highlighted that many cultivation experiments resulted in nothing because they failed to take economic aspects into consideration. Studies have revealed that black plum has an important market [13], that keeps expanding and this represents an incentive for its cultivation and conservation [40]. The species was also mentioned as present in at least 20% of traditional agroforests, from a countrywide survey in Benin [21]. In these conditions, cultivation of black plum is likely to be successful. In fact, *V. doniana* has the highest use value among the wild tree resources of importance to local livelihoods, especially for women and illiterate people, who tend to value it more than any other tree species. We also found that people with higher educational level seemed to value cash tree crops more highly than others, namely *E. guineensis, A. occidentale,* and *T. grandis* by decreasing order of importance. This could be an indication of the erosion of local value among the more “westernized” households. The educational elite classify resources almost solely based on market value of species, completely ignoring the social and cultural values associated with them. Another consideration is that they have less contact with wild resources in nature. Migrants also seemed to value *V. doniana* and *P. biglobosa* more highly than autochthons to whom the cash tree crop *A. occidentale* is the top most important. In fact, in the local land tenure systems migrants who are not owners of land have no right to plant perennial species. However, they have free access to wild resources in the nature, a fact which could explain this high value they attributed to the wild species. Overall, these variables are significantly important and should be carefully taken into account when selecting stakeholders in wild resource conservation programmes.

### Uses, management and conservation strategies

Results clearly indicated that black plum is a multipurpose species, a characteristic of most NWFPs [41]. The inventoried uses confirmed the current state of knowledge on the species as reported by previous studies [12,16,40,42]. The high IAR values confirmed that knowledge of these uses is well distributed among user communities and the species is important in rural livelihoods. Exploitation of the species is profitable for all age groups, but more so for women than men. This is explained by the fact that men are most involved at the primary stage of exploitation, as primary collectors; while women are more involved in the wholesale and retail of processed leaves and fruits. By the law of profit distribution along the market chains of NWFPs, wholesalers and retailers (involving mainly women) obtain the bigger profit [43]. Species of this type are classified under the “*women goods*” categories of NWFPs [44].

Overall, juvenile trees are more vulnerable to disruption caused by destructive harvesting practices and wild fires. Therefore, much of any regeneration dies out at the end of the dry season. Overharvesting of leaves reduces fruiting ability of the overharvested trees, and this coupled with fruit harvesting obviously reduces the possibility of regeneration from seed. All these represent important threats to survival of wild populations. Nonetheless, the current level of roots and bark harvesting for medicinal purposes is low and does not add significantly to existing threats.

The analysis of access to resources of different landscapes indicated an incipient privatization of *V. doniana* trees occurring in farmlands and fallows. In fact, trees that are spared during land clearance for agriculture become the property of the farm owner. This tenurial system enhances conservation, as harvesting is better scheduled and competition with other harvesters is limited. On common lands (woodlands and savannas) trees are often cut down to harvest the leaves when they are too difficult to climb. The trunk and branches left out are then used to make charcoal or fuel wood. In farms, when the owner grants a usufruct right to a person to harvest trees on his or her fields, that person is not allowed to cut *V. doniana* trees. These kinds of measures protect the species.

From our data, local communities did not report any variants of *V. doniana* that can be subject to artificial selection in the sense of Blancas *et al.* [3]. However, referring to Blancas *et al.* [3]’s classification of the different management types, we distinguished the following categories which are considered as *in-situ* actions:

i) “*Gathering without recognition of variants*”. Respondents did not report any selective harvesting of *V. doniana* resources. No variants were reported within the species.
ii) The species is also “*tolerated without recognition of variants*”, whereby stands were spared in farms but without any special tendering. This tolerance did not consider variants.
iii) For the very few cases of regeneration reported by local communities (5.61%), the species is “e*nhanced without recognition of variants*”. People tried to regenerate the species in order to increase its abundance and subsequent availability, and this was not based on variants.
iv) The species is “*protected without recognition of variants*”. This protection mainly happens in farms where people spare *V. doniana* trees during land clearance and weeding, without any distinction of variants. No additional care is provided.

Other categories such as “*seed sowing*” and “*propagation of vegetative parts*” described by Blancas *et al.* [3] were observed at a very low proportion (3.93% used seeds and 1.68% used stem cuttings). These two categories were classified as *ex-situ* management by Blancas *et al.* [3]. However, based on the definitions of *in-situ* and *ex-situ conservation* by the CBD [34], we rather identified them as *in-situ* management, as these activities entirely happened in farms in the case of *V. doniana*.

Furthermore, future horticultural investigations should tackle the issue of keeping trees at a size and height that facilitate harvesting. This was further emphasized in the conditional inference model where it clearly appeared that some farmers put this as a condition before they engage into the cultivation of the species. Those farmers would prefer a shorter *V. doniana* tree which can be easily harvested.

Men have better knowledge of tree phenology (especially the fruiting period) than women, although women were the most involved in *V. doniana* exploitation. This is explained by the fact that men are the most involved in the primary collection of the resources and thus are more in contact with the species in its natural habitat. In addition, the autochthons have better knowledge of the biology of the species than migrants. Explanation to this might be that the autochthons have been in contact with the species for long time and knowledge is transmitted through generations. This indicates the importance of indigenous people’s ecological knowledge on NWFPs. It was also found that local knowledge of people is eroded with schooling. This negative effect was also reported by Chukwuone [10] where schooling had negative effect on involvement of local people in NWFPs cultivation in Nigeria. At the current state, conservation initiatives are very poor in the study area, with very limited on-farm sparing of trees during land clearance for agriculture. This has been reported by N’Danikou *et al.* [12] and Dadjo *et al.* [40]. With regard to this and taking into account the pace with which the resource is harvested, wild populations of *V. doniana* are undoubtedly under threat. Based on the current level of local conservation initiatives, *V. doniana* falls under steps 1 and 2 of the domestication process [6,35]. Step 1 is characterized by wild plants maintained on farms during land preparation (e.g. clearance, burning and weeding) due to their proven utility and regular need, and their scarcity within household’s compound limits. These spared plants are regularly observed to learn about their reproductive biology. In Step 2, farmers start giving more care to the protected plants (e.g. weeding, protection against animal damage) for their survival and their normal growth; a sort of ownership on the plants starts [35]. It is important to note that up to Step 2, no modification of plants has occurred due to selection; but plants just incidentally co-evolved to adapt to the disturbance imposed by human activities in the environment [6].

### Motivations for cultivation and conservation of wild populations of *V. doniana* by user communities

Based on the current demand for *V. doniana* products and the intensity of the harvest, cultivation would be the best option to sustain the species and also the livelihoods of the different user groups. However, *V. doniana* is still at the earlier stage of the domestication process as indicated above and is still intensively collected from the wild. Thus, it is vital to understand what could motivate resource users to engage in cultivation of the species. From our study and based on the conditional inference tree statistical approach, four main variables have important effects on the decision-making process to cultivate *V. doniana* in the surveyed communities. This is partly consistent with previous studies in this domain [12,21]. First, an important proportion of users have expressed their willingness to cultivate the species for its importance in household food consumption and income. The second important parameter to be considered is schooling and literacy. Findings indicated that the use value of *V. doniana* to people declines with schooling. In this respect, awareness needs to be raised to sensitize elite classes. A third variable weighting in the cultivation of the species is the land tenure. Tenants were less likely to accept cultivation. In fact, the land tenure system in southern Benin and in any other parts of the country does not grant the tenants the right to plant trees [25]. Perennial species and most often trees are used to mark ownership of land. Thus, individuals with no property right on land may be willing but not ready to invest into cultivation of the species. At fourth and the most important, tenure size is key to the decision to cultivate and to maintain the species *in-situ*. People with larger parcels of land were eager to allocate bigger plots for the agroforestry of the species and were more likely to consider cultivation. Where land is available gender is an important factor in the likelihood for *V. doniana* cultivation. Although women’s use value of the species was higher than men’s, the men were more likely to plant the species. This could in fact be explained by the tenure system in which women have less access to land. There are also other variables such as the importance of wild-harvesting, the location and the cost of seedlings that were considered by people in their decision making. The threshold of XOF 650 (USD 1.44) found is well reflected in the positive relationship between the cultivation opportunity ratio (COR) and the likelihood to cultivate. All those who proposed a seedling acquisition cost below this threshold were not willing to cultivate.

### Implications for management of overharvested wild resources

Several wild resource conservation programmes have failed around the world due to the lack of clear understanding of local drivers for success [10]. Gepts *et al.* [4] indicated that the success of conservation through use and restoration of biodiversity requires continued and increased research, and policies. Our findings pinpoint the main socioeconomic factors that trigger the success of conservation and management of threatened wild harvested plant resources with high economic value. Motivated local communities should be at the heart of conservation management, and we demonstrate that there is considerable willingness to engage in conservation of a locally threatened economically significant wild species. The availability of land was the most important element in our study with gender and lack of awareness of the importance of local foods among the elite class. The determined affordable cost of seedlings for farmers is a good entry point for nursery development. The negative values found for the cultivation opportunity ratio is also a good indication of the willingness of people to grow *V. doniana* in their farmlands and conserve wild populations. This means that less effort would be needed to get this category of participants involved.

The present study also encompasses the principle of complementarity between biological diversity conservation strategies. In fact, each of the elements treated in this paper (i.e. cultivation and *in-situ* conservation) have both positive and negative outcomes for biodiversity conservation, which need to be discussed. Plant cultivation can result in important setbacks if not well managed, despite its important advantages [45]. A plant cultivation programme that is not sustainably managed could lead to genetic drift and important biodiversity loss caused by high selection and breeding of superior lines. In the case of wild-harvested species such as *V. doniana* which have not been evaluated genetically, it is necessary to conserve wild populations in their natural habitats and in *ex-situ* genebanks (if possible) while they are undergoing domestication; in order to safeguard the gene pools in the species for future breeding purposes. Besides, there are examples of successful domestication of plant species whereby this undertaking has increased diversity. For instance in the case of maize which, despite the long history of selection and genetic improvement it has undergone, developed a huge diversity of cultivars. This was possible thanks to the ecological diversity of agricultural landscapes, which promoted the spread of cultivars and their adaptation to a diversity of agroecological niches [46], but also due to conservation of ancestors in the wild and *ex-situ* in genebanks. In the other hand, despite its positive effect in safeguarding diversity, *in-situ* conservation can also have adverse effects. In fact, populations that are conserved *in-situ* undergo natural selection. Although this favours coevolution and adaption to the changing environment, a fact which is sought in breeding for adaptive traits, a great diversity of genes is lost during the process. The weakest individuals which cannot survive changes that occur in the natural environment, though they may possess other useful traits for future breeding purposes, disappear and new genetic material of surviving individuals is established. Taking all the above into consideration, Article 9 of the Convention on Biological Diversity (CBD) advocates that *in-situ* conservation, as far as possible and as appropriate, be complemented by *ex-situ* measures [34].

### Implications for research and development

From our findings, there is an avenue for domestication of *V. doniana* and conservation managers and horticulturalists should consider means to alleviate agronomic constraints such as keeping the plants at a shape and height to facilitate harvesting. Based on intrinsic characteristics and behavior of plants in the agricultural environment, domestication of *V. doniana* would just require adaptation to the agricultural environment [5]. Early results of horticultural trials indicated that the species responds very well in agroforestry systems (authors’ unpublished data). In the debate on where and when domestication of wild species takes place it is often argued that the key determinant is the ecological but also the intrinsic characteristics of the wild plant or animal species which rendered them suitable for domestication which downplays the cultural, social and technological prowess of the hunter-gatherers themselves [4]. The authors, however, argue that in addition to biological and ecological traits, there are socio-cultural and economic factors influencing the genetic make-up of farmer bred varieties. In this regard, our findings make an important contribution to practical NWFP conservation. They are particularly relevant for the future of ethnobotanical studies aimed at providing advice to conservation management. The decision making process in the co-management or participatory natural resource management frameworks should use holistic approaches that integrate the social, cultural and economic dimensions of the local people in relation to wild plants use. Selection of participants in conservation and domestication programmes should be based on criteria that help to recruit members that are most likely to take actions to good ends. The approach used in the current research is simple and can be applied to any wild harvested resource similar to black plum. Nonetheless, future investigations of this type should dig further into the characteristics of locations that might make their inhabitants more active in wild resource cultivation. Chukwuone [10] identified the distance to reach harvesting sites and level of resource depletion. The effect of market access should also be further investigated. In our study access to market was not investigated as a variable, as all villages have relatively easy access to a market. Another important area of investigation is the analysis of the value chain of the species and the role of the stakeholders in a scenario of an organized commercial production of the species. Final, it is worthwhile noting the limitation of testing the relationship between presumed single explanatory variables and a response variable which is under the control of several factors. In our study the conditional inference tree model seemed most robust to unveil the rationale behind the decision making process about cultivation and *in-situ* conservation of species. This calls for caution when drawing general conclusions from inferential statistics on a phenomenon which is under the control of complex social, biological and environmental factors [47].

## Conclusion

This study highlighted factors that trigger cultivation and *in-situ* conservation of wild harvested resources, with *V. doniana* as a case study. The species is thought to be declining at a high rate and all informants agreed that there is a need for action. Overall, we demonstrated that cultivation of the species is possible and eight main drivers were identified. These included land tenure, size of land holding, gender, cultivation opportunity ratio, schooling and literacy, resource availability measured through the importance of wild-harvesting, affordability of seedlings, and location. We also demonstrated that the Cultivation Opportunity Ratio is an important indicator for quick assessment of the likelihood of farmers to engage into cultivation and conservation of the species. The findings come in support to the agronomic investigations that are being developed for cultivation of the species. Only the combination of on-farm management and *in-situ* conservation would help take pressure off wild populations to safeguard genetic diversity in the species and enhance livelihoods of the various user groups.
